# SWITCH: a dynamic CRISPR tool for genome engineering and metabolic pathway control for cell factory construction in *Saccharomyces cerevisiae*

**DOI:** 10.1186/s12934-017-0632-x

**Published:** 2017-02-08

**Authors:** Katherina García Vanegas, Beata Joanna Lehka, Uffe Hasbro Mortensen

**Affiliations:** 1 Department of Biotechnology and Biomedicine, Technical University of Denmark, Søltofts Plads, Building 223, Room 208, 2800 Kgs. Lyngby, Copenhagen, Denmark; 2 Department of Science and Environment, Roskilde University, Universitetsvej 1, 4000 Roskilde, Denmark

**Keywords:** CRISPR tool, Genome engineering, Metabolic pathway control, Cell factory, *Saccharomyces cerevisiae*

## Abstract

**Background:**

The yeast *Saccharomyces cerevisiae* is increasingly used as a cell factory. However, cell factory construction time is a major obstacle towards using yeast for bio-production. Hence, tools to speed up cell factory construction are desirable.

**Results:**

In this study, we have developed a new Cas9/dCas9 based system, SWITCH, which allows *Saccharomyces cerevisiae* strains to iteratively alternate between a genetic engineering state and a pathway control state. Since Cas9 induced recombination events are crucial for SWITCH efficiency, we first developed a technique TAPE, which we have successfully used to address protospacer efficiency. As proof of concept of the use of SWITCH in cell factory construction, we have exploited the genetic engineering state of a SWITCH strain to insert the five genes necessary for naringenin production. Next, the naringenin cell factory was switched to the pathway control state where production was optimized by downregulating an essential gene *TSC13*, hence, reducing formation of a byproduct.

**Conclusions:**

We have successfully integrated two CRISPR tools, one for genetic engineering and one for pathway control, into one system and successfully used it for cell factory construction.

**Electronic supplementary material:**

The online version of this article (doi:10.1186/s12934-017-0632-x) contains supplementary material, which is available to authorized users.

## Background

Fermentation offers alternative production of a wide variety of compounds ranging from primary- and secondary metabolites to enzymes and therapeutic proteins. Hence, cell factories may replace productions depending on polluting resource-demanding petro-chemistry and/or productions where natural bio-production is difficult, unstable, and costly. However, development of new economically viable cell factories is often labor intensive, technically difficult, and time consuming. New tools to speed up and simplify cell factory construction are therefore highly desirable as they pave the way for sustainable production of high-quality and cost-effective products for the benefit of the environment and the consumers [1, 2].

Although efficient methods for genome engineering of popular cell factories like *Escherichia coli* (*E. coli*) and *Saccharomyces cerevisiae* (*S. cerevisiae*) have been available for decades, strain development and optimization are still time consuming processes requiring a diverse range of multidisciplinary techniques, expertise and practical skills. One important reason for this is that it is rare that one or a few genetic engineering steps lead to formation of an efficient cell factory. Rather extensive multi-step metabolic engineering and/or tedious improvements via classical mutagenesis or evolution based methods are required to achieve economically attractive titers. Construction time may therefore be reduced by developing general techniques to speed up the experimental cycle for strain construction. Here we address this possibility using CRISPR/Cas9 derived technologies for construction of yeast-based cell factories.

Recently, CRISPR/Cas9 based technologies have been introduced as advanced and flexible tools for metabolic engineering that may radically speed up cell factory construction. For example, it is well documented that Cas9, due to its ability to introduce specific RNA guided DNA double strand breaks (DSBs), can be used to greatly stimulate homologous recombination (HR) based genetic engineering at specific loci [3, 4]. Accordingly, Cas9 sets the stage for modifying several target genes, or introducing multiple genes, in single transformation experiments [5–11]. With Cas9, genetic engineering is so efficient that accompanying selection markers are not required. This is important, as iterative gene targeting can then be performed without need for marker recycling. Moreover, in most cases industrial producer strains do not possess e.g. antibiotic resistance marker genes; and engineering can therefore be performed in genetic backgrounds that are closer to production strains [12]. Another feature of CRISPR/Cas9 based technology is its ability to act as a target specific synthetic transcriptional regulator. In this case, the endonuclease inactive variant dCas9 is targeted to relevant promoters via a guide RNA (gRNA) and mediates up- or downregulation of target genes. For example, if dCas9 binds to a promoter or in an open reading frame, ORF, it may act as a repressor. In this case, the gRNAs responsible for the interactions are referred to as interference gRNAs. In contrast, by fusing dCas9 to a regulatory domain (RD), e.g. VP64, it may act as an activator [13–15]. Recently, other CRISPR associated nucleases with different gRNA binding- and endonucleolytic properties have been presented in the literature [16, 17] and these nucleases serve as alternatives to Cas9 for genetic engineering. In this paper, we refer to Cas9 and other CRISPR associated nucleases as CasX.

**Fig. 1:**
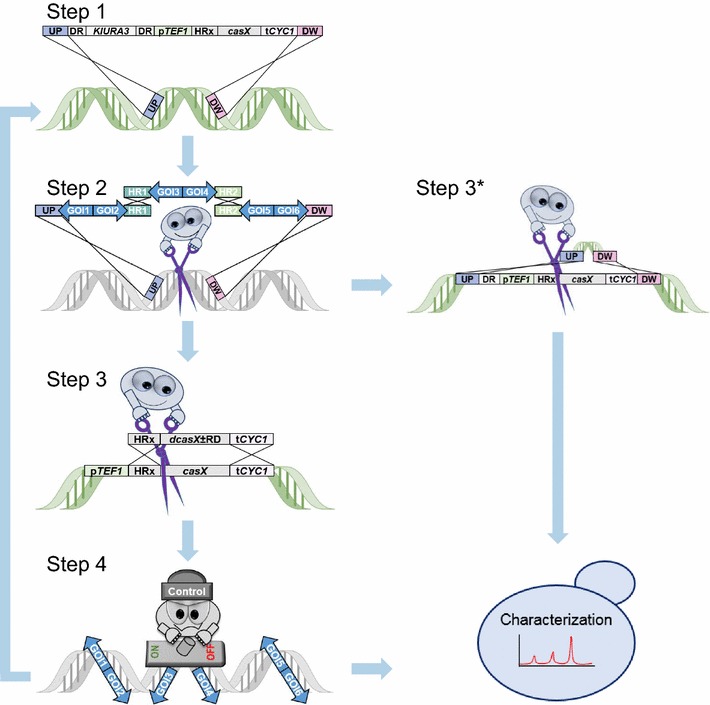
The SWITCH strategy for cell factory construction and optimization. *Step 1* The genomic engineering state is created by integrating *casX*. Note that a direct repeat flanks the *KlURA3* marker allowing it to be recycled via direct repeat (DR) recombination. *Step 2* In one transformation event several genes of interest (GOI) are simultaneously and marker-less integrated by the unified support of assembler and CasX. *Step 3* The genomic engineering state is switched into the regulatory state, when CasX is directed to cleave its own gene sequence. The rescue DNA fragment contains either a codon optimized *dcasX* or a *dcasX* fused with a regulatory domain (*dcasX* ± RD) flanked by regions that are homologous to the integrated *casX* cassette. Alternatively, step 3* if the strain is finalized in step 2, the locus containing *casX* can be restored to wild type by the assistance of CasX and a rescue fragment containing the locus sequence. *Step 4* In the regulatory state the regulator protein (dCasX or dCasX-RD) can be used to target both endogenous and heterologous GOI. Finally, after both step 3* and 4 the newly created cell factory can be characterized as part of a metabolic engineering cycle

Both the genetic engineering and gene regulatory aspects of CRISPR/Cas9 have advantageously been applied in metabolic engineering strategies for cell factory construction and optimization. We have therefore developed SWITCH that allows a strain to change between CasX mediated genetic engineering and dCasX mediated regulation states in cycles where switching is based on efficient CasX induced recombination events, see Fig. 1. One engineering/regulatory cycle is achieved by one specific CasX species; a second cycle is achieved by another species, and so on. In this way a cell factory can either be developed by an optimization cycle where the strain alternates between states where it can be genetically engineered or states where different levels of gene regulation can be implemented. In the present paper, we use Cas9 and dCas9 variants to demonstrate proof of principle of SWITCH by implementing and tuning the pathway for naringenin (NG), a valuable flavonoid possessing strong antioxidant and anti-inflammatory activities in vitro and in vivo [18], as a model system.

## Results and discussion

### SWITCH: a CRISPR based system for rapid genetic engineering and pathway tuning

A full cycle of SWITCH requires four steps: (1) specific integration of *casX*, (2) CasX mediated genetic engineering, (3) replacement of *casX* for *dcasX*, (4) specific metabolic tuning mediated by dCasX. In SWITCH *casX* and *dcasX* gene variants are integrated into well-characterized genomic loci exploiting a gene-expression platform we have previously developed for *S. cerevisiae* [19, 20]. The platform currently contains 15 integration sites and can therefore support 15 SWITCH cycles. In the first step of SWITCH, *casX* is stably integrated into one of the specific loci in the yeast expression platform producing a strain, which is in the genetic engineering state (Step 1, Fig. 1). Next, gRNA mediated genetic engineering can be iteratively performed. For example, an entire pathway may be establish by inserting the individual genes one by one using multiple rounds of transformation, or in one or a few steps by using e.g. the assembler technology (Step 2, Fig. 1) [21]. When genetic engineering is complete, *casX* can be either eliminated if the strain is ready for characterization (Step 3*, Fig. 1), or, substituted for a gene encoding a dCasX variant, hence, setting the stage for pathway regulation (Step 3, Fig. 1 and Additional file 1: Figure S1 for details). In both cases, recombination is catalyzed by CasX itself and only requires that the strain is co-transformed with a plasmid encoding a gRNA directing the CasX nuclease to the *casX* gene and a gene-targeting substrate containing the *dcasX* or *dcasX*-*RD* sequence or a sequence that restores the *casX* integration site. Repair of the resulting DNA DSB in *casX* using the gene-targeting substrate as repair template results in the desired replacement of *casX* with *dcasX or dcasX*-*RD;* or in restoration of the casX integration site if pathway characterization is the next step. After completing step 3 a plasmid-free strain is selected and then transformed with a new gRNA encoding plasmid setting the stage for step 4. In the transformed cells the gRNA directs dCasX-RD to gene(s) that are targeted for up- or down-regulation (Step 4, Fig. 1). The cycle can be repeated by exploiting a new *casX/dcasX* variant with different gRNA binding properties in each cycle.

#### Testing and optimizing the genetic engineering state of SWITCH

We first established Step 1 by integrating a *cas9* gene (codon optimized for human cells) [22] in strain S-0 (see Table 1). Specifically, *cas9* under the control of the *TEF1* promoter was inserted into the X-3 integration site of our yeast expression platform [20] using a *URA3* marker for selection. Transformants were easily obtained and twelve clones were randomly picked and tested for the presence of *cas9* at the X-3 site. All transformants contained correctly integrated *cas9* genes as judged by a PCR based test (Additional file 1: Figure S2). For one of these transformants, the *URA3* marker was eliminated by direct repeat recombination, and the resulting strain S-1, was used in further experiments.

**Table 1:** Strains used in this work

Strains	Genotype	Source
PJ69-4	*MATa trp1*-*901 leu2*-*3,112 ura3*-*52 his3*-*200 gal4D gal80D LYS2::GAL1*-*HIS3 GAL2*-*ADE2 met2::GAL7*-*lacZ*	[28]
PJ69-4 S-1	*MATa trp1*-*901 leu2*-*3,112 ura3*-*52 his3*-*200 gal4D gal80D LYS2::GAL1*-*HIS3 GAL2*-*ADE2 met2::GAL7*-*lacZ X*-*3::pTEF1*-*hcas9*-*tCYC1*	This study
PJ69-4 S-2	*MATa trp1*-*901 leu2*-*3,112 ura3*-*52 his3*-*200 gal4D gal80D LYS2::GAL1*-*HIS3 GAL2*-*ADE2 met2::GAL7*-*lacZ X*-*3::pTEF1*-*dcas9*-*VP64*-*tCYC1*	This study
S-0	*MATα Δura3 Δpad1 Δfdc1 Δleu2 Δaro10*	This study
S-1	*MATα Δura3 Δpad1 Δfdc1 Δleu2 Δaro10 X*-*3::pTEF1*-*hcas9_tCYC1*	This study
S-2	*MATα Δura3 Δpad1 Δfdc1 Δleu2 Δaro10 X*-*3::pTEF1*-*hcas9*-*tCYC1 XI*-*2*::[*pTDH3*-*AtPAL2*-*tPGI1 TEF2*-*C4H L5 ATR2*-*tCYC1 pPGK1*-*HaCHS*-*tENO2 pTEF1*-*PhCHI*-*tFBA1 pPDC1*-*At4Cl2*-*tTDH2*]	This study
S-3	*MATα Δura3 Δpad1 Δfdc1 Δleu2 Δaro10 X*-*3::pTEF1*-*dcas9*-*tCYC1 XI*-*2*::[*pTDH3*-*AtPAL2*-*tPGI1 TEF2*-*C4H L5 ATR2*-*tCYC1 pPGK1*-*HaCHS*-*tENO2 pTEF1*-*PhCHI*-*tFBA1 pPDC1*-*At4Cl2*-*tTDH2*]	This study
S-4	*MATα Δura3 Δpad1 Δfdc1 Δleu2 Δaro10 XI*-*2*::[*pTDH3*-*AtPAL2*-*tPGI1 TEF2*-*C4H L5 ATR2*-*tCYC1 pPGK1*-*HaCHS*-*tENO2 pTEF1*-*PhCHI*-*tFBA1 pPDC1*-*At4Cl2*-*tTDH2*]	This study

Efficient Cas9 mediated marker-free genetic engineering is crucial for SWITCH cell factory construction. Since specific Cas9 nuclease efficiency depends on the sequence of the protospacer [23, 24], it is important to choose efficient gRNAs. As unrepaired DNA DSBs are lethal in *S. cerevisiae* [25, 26] we envisioned that the efficiency of a given gRNA in guiding Cas9 to a specific locus will be reflected in cell death in the absence of a repair template.

**Fig. 2:**
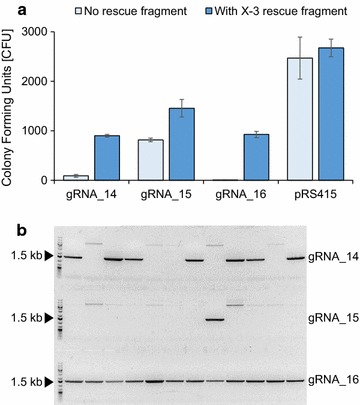
Efficient setup for SWITCH. **a** Screening for gRNAs that efficiently targets Cas9 to the *cas9* gene, using TAPE, see text for details. **b** Confirmation of X-3 locus restoration by genomic PCR of 12 clones randomly selected amongst the transformants obtained in the presence of a X-3 rescue fragment. The presence of a 1.5 kb PCR fragment indicates that the X-3 locus has been restored

To explore this idea, we individually transformed three centromere-based *LEU2* plasmids (see “Methods”) encoding three different gRNAs, each of which matches different sequences in X3::*cas9,* as well as a control plasmid pRS415 into S-1 strains (Fig. 2a). Despite that we used identical concentrations of the four plasmids, the numbers of transformants obtained with the plasmids encoding gRNA_14, gRNA_15, and gRNA_16 were reduced 27-, 3-, and 494-fold, respectively, as compared to the number obtained with pRS415; and these differences were all significant (*p* values <0.05). Moreover, the numbers of transformants obtained with gRNA_14 and with gRNA _16 were significantly different from the number obtained with gRNA_15 (*p* values <0.005). In contrast, the numbers of transformants obtained with all four plasmids individually transformed into strain S-0, which does not contain the *cas9* gene, were identical (*p* value >0.26), see Additional file 1: Figure S3. Together these results indicate that the three plasmids encoding gRNAs induce cell death in the S-1 strain by forming Cas9 mediated DNA DSBs and that amongst the three gRNAs, gRNA_14 and gRNA_16 may be the best candidates for efficient DNA editing.

Next, we explored whether lethal gRNA-Cas9 induced DNA DSBs at *cas9* in the X-3 locus could be rescued by including a linear X-3 rescue fragment in the transformation mixture. Indeed, we observed that the numbers of transformants obtained with gRNA_14, gRNA_15, and gRNA_16 plasmids in S-1 strains could be significantly increased by 10-, 1.8-, and 185-fold (*p* values <0.05), respectively, (Fig. 2a). In contrast, when S-1 strains were transformed with the control plasmid in the absence or presence of the X-3 rescue fragment, the numbers of transformants were not significantly different (*p* value 0.59) showing that the X-3 rescue fragment alone does not increase the transformation efficiency. These results strongly indicate that the X-3 rescue fragment can serve as a template for HR mediated repair of gRNA-Cas9 induced DNA DSBs at *cas9* during transformation.

Successful repair of gRNA-Cas9 induced DNA DSBs at *cas*9 using the X-3 rescue fragment as template restores the X-3 locus at the expense of the *cas9* gene. To test how efficient the three *cas9* specific gRNAs mediate this reaction, we analysed twelve randomly selected transformants from each of the three co-transformation experiments described above by PCR. Our results above predict that gRNA_15 is the worst protospacer and that gRNA_14 and gRNA_16 are the more efficient protospacers. In agreement with this, only one out of twelve transformants obtained with gRNA_15 and the X-3 rescue fragment, contained wild-type X-3. In contrast, seven and twelve out of twelve transformants obtained with gRNA_14 and gRNA_16, respectively, in the presence of the X-3 rescue fragment contained wild-type X-3 (Fig. 2b). Hence, with this set of experiments we have developed a technique to assess protospacer efficiency, which we call TAPE, and used it to make a highly efficient setup for a switch that restores the X-3 locus by eliminating *cas9* in X-3. Based on these results, we used gRNA_16 to replace *cas9* with *X*-*3*, *dcas9* or *dcas9*-*RD* in all subsequent experiments.

To investigate whether SWITCH, in the genetic engineering state, can be successfully used for construction of a cell factory, step 2 in Fig. 1, we used TAPE to identify a gRNA that efficiently targets the expression site XI-2. To this end, three plasmids encoding different XI-2 specific gRNAs were transformed into S-1 (Additional file 1: Figure S4a). TAPE results indicated that gRNA_23 was the most efficient candidate since a plasmid encoding this species produced a significantly lower number (>77-fold reduced) of transformants as compared to those obtained with plasmids encoding gRNA_13, gRNA_22, or no gRNA (all *p* values <0.05), see Additional file 1: Figure S4b.

**Fig. 3:**
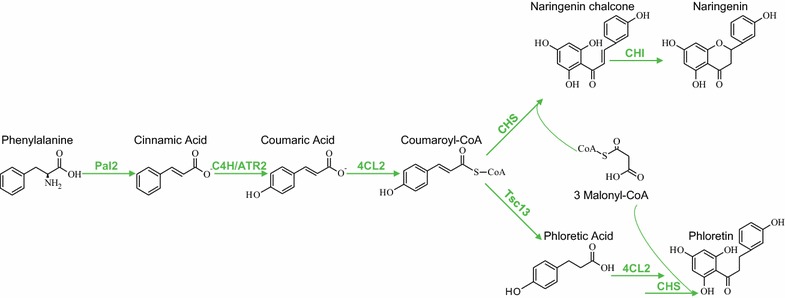
Pathway reactions involved in production of naringenin and the by-product phloretin from phenylalanine. *Pal2* phenylalanine ammonia-lyase 2, *C4H/ATR2* cinnamate 4-hydroxylase/NADPH-cytochrome P450 reductase 2, *4CL2* 4-coumarate:coenzyme A ligase 2, *CHS* naringenin-chalcone synthase, *Tsc13* trans-2-enoyl-CoA reductase (NADPH), *CHI* chalcone isomerase

Using Cas9 induced DNA DSBs to induce marker-free assembler integration [11], we next attempted to integrate all five genes that are required for NG production [27] into S-1 (Fig. 3). Accordingly, three assembler fragments containing the relevant genes and targeting sequences were simultaneously transformed into S-1 where they were fused and integrated into XI-2 by HR (Additional file 1: Figure S5). As expected for low efficiency protospacers, no (*p* value 0.32) or a low (1.9-fold; *p* value <0.05) increase in number of transformants was observed when gRNA_22 and gRNA_13 were transformed into S-1 strains in the presence of assembler fragments, as compared to the corresponding numbers obtained in their absence. In contrast, with gRNA_23, which TAPE identified as a highly efficient protospacer, the transformation efficiency was increased more than 128-fold (*p* value <0.005) when the assembler fragments were included in the transformation reaction as compared to the corresponding numbers obtained without the assembler fragments (Additional file 1: Figure S4b). These results strongly indicate that the assembler fragments were efficiently fused by HR and used as a template for repair of the DNA DSBs induced by Cas9-gRNA_23. In support of this, 24 randomly picked colonies from this experiment all contained the five NG genes integrated into XI-2 as judged by PCR analysis (Additional file 1: Figure S6a and b). Finally, all strains were subjected to metabolite analysis. In agreement with the PCR test, the HPLC- UV/DAD analysis showed that all 24 strains produced NG (Additional file 1: Figure S6c). One random transformant was named strain S-2 and used to enter step 3.

#### Establishment of an efficient *cas9*–*dcas9* gene-swap procedure

**Fig. 4:**
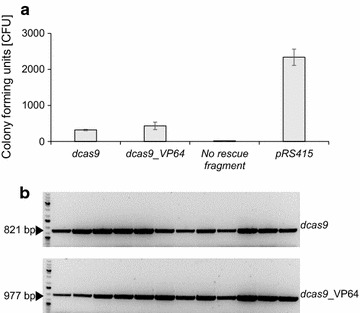
By SWITCH, the *cas9* gene can be efficiently replaced by *dcas9* and *dcas9_VP64* genes. **a** *dcas9* and *dcas9_VP64* fragments efficiently rescue DNA DSBs formed by Cas9-gRNA_16 in the *cas9* gene. **b** Confirmation of SWITCH gene replacements by PCR of 12 randomly selected clones co-transformed with gRNA_16 and (*top*) the *dcas9* rescue fragment and (*bottom*) the *dcas9_VP64* fragment. Diagnostic PCR fragments for *dcas9* and *dcas9_VP64* are 821 and 977 bp, respectively

A key step, step 3 of SWITCH, is the ability to switch the host strain from a Cas9 genetic engineering state to a dCas9 regulatory state in a simple and efficient manner. We therefore tested whether Cas9-gRNA_16, which efficiently targets *cas9* (see above and Additional file 1: Figure S1), can be used to catalyze marker-free swapping of *cas9* for *dcas9* (codon optimized for *S. cerevisiae*). As expected for an efficient protospacer, S-1 derived transformants were significantly easier to obtain (*p* values <0.05) with the plasmid encoding gRNA_16 in the presence of linear marker-free repair fragments (*dcas9* or *dcas9*-*VP64*) than in the absence of these fragments. In fact, the transformation efficiency with the plasmid encoding gRNA_16 was increased 13-fold and 17-fold in co-transformation experiments that included the repair fragments *dcas9* or *dcas9*-*VP64*, respectively, as compared to the corresponding transformations that did not include repair fragments (Fig. 4a). For both gRNA_16 co-transformation experiments, twelve transformants were analysed by PCR and the results showed that in all cases *cas9* has been replaced with the sequence contained in the repair fragment e.g. either with *dcas9* or with *dcas9*-*VP64* (Fig. 4b). Besides supporting that our system to evaluate gRNA proficiency is robust, these results demonstrate that we have developed an efficient *cas9*–*dcas9/dcas9*-*RD* gene-swap procedure with an efficiency approaching 100%.

#### Synthetic dCas9 derived transcription factors for SWITCH

We then investigated the possibility of using dCas9 and dCas9-VP64 as synthetic transcription factors (STFs) in our SWITCH setup using gRNAs identified by TAPE. For this purpose we employed a two-hybrid strain PJ69-4 [28] where the native *ADE2* gene has been replaced with a synthetic reporter gene *pGAL2::ADE2*, and where the gene encoding the Gal4 transcription factor has been deleted. We next designed STFs composed by gRNAs matching the *pGAL2* promoter and dCas9-VP64 and tested their ability to activate the synthetic reporter gene in two different ways. Firstly, Ade2 activity will allow the strains to propagate on medium that does not contain adenine. Secondly, when Ade2 activity is limiting, colonies will appear red on medium containing limiting amounts of adenine due to accumulation of a metabolic intermediate in the purine biosynthesis [29]. In contrast, successful activation of *pGAL2::ADE2* by dCas9-VP64 activity will result in white or less red colonies on such a medium.

**Fig. 5:**
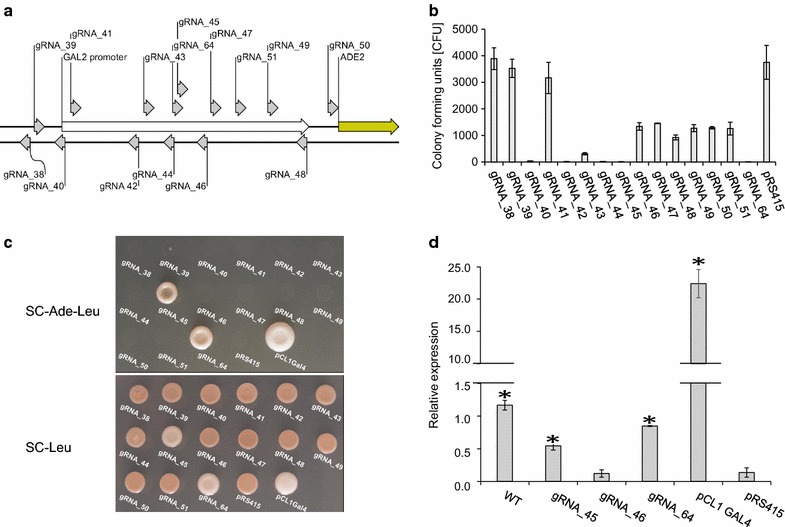
Implementing and exploiting the regulatory state of SWITCH. **a** Localization of the 15 gRNAs tested for *GAL2* promoter activation. **b** Screening of the efficiency of the 15 gRNAs to guide Cas9 to the *GAL2* promoter using TAPE. **c** Evaluation of *ADE2* expression in colony patches on both SC-Ade-Leu and SC-Leu solid media. **d** Determination of *ADE2* transcript levels relative to *ACT1* by qRT-PCR in selected strains as indicated. *Stars above columns* indicate strains with an *ADE2* expression level, which is significantly different (*p* values <0.005) from the corresponding level obtained from a control strain harboring pRS415

First, we used TAPE to identify gRNAs that efficiently target Cas9 to *pGAL2*. Accordingly, 15 plasmids, each encoding different specific sequences matching *pGAL2* (Fig. 5a), were tested for their ability to transform a *cas9::X*-*3* strain (PJ69-4 S-1) that also harbors the *pGAL2::ADE2* assay. With ten of the plasmids more than 200 transformants were obtained (Fig. 5b). For the remaining five plasmids, significantly less colonies were obtained (>8.9 fold reduced; all *p* values <0.05) indicating that the gRNAs encoded by these latter plasmids result in severe Cas9 induced cell death.

Next, PJ69-4 S-1 was switched from the genetic engineering state to the regulatory state by co-transformation with a plasmid encoding gRNA_16 and a *dcas9*-*VP64* repair fragment to form strain PJ69-4 S-2. All plasmids encoding gRNAs for targeting Cas9 to *pGAL2* in PJ69-4 S-1 were then individually transformed into PJ69-4 S-2.

Transformants obtained with the 15 plasmids were randomly picked and tested in a spot assay on solid SC-Leu-Ade medium (Fig. 5c). PJ69-4 S-2 transformed with a control plasmid (pCL1) encoding Gal4 [30] grew on this medium and formed a colony patch, but the ten transformants harboring a plasmid encoding gRNAs that were poor gRNAs, as judged by TAPE, did not propagate. In contrast, amongst the five transformants encoding efficient gRNAs, two grew on this medium and formed colony-patches. The fact that only two of the five efficient gRNAs support growth on this medium may reflect that the binding position of STFs on *pGAL2* is important for inducing transcription of *ADE2*. To this end we note that the two proficient STFs bind to the same region of *pGAL2*, (see Fig. 5a). Similar results were observed in attempts to activate the *CYC1* promoter by STFs [13].

Next, we tested the 15 transformants in a spot-assay on solid SC-Leu (Fig. 5c). The ten transformants harboring a plasmid encoding gRNAs that were poor gRNAs all produced red colony-patches. Of the five transformants that harbored plasmids encoding efficient gRNAs, the three that did not propagate on solid SC-Ade-Leu medium also formed red colony patches as expected. However, the two transformants that did propagate on solid SC-Ade-Leu formed colony-patches that were pink in agreement with active gene expression from *ADE2.* We note that the control strain expressing *GAL4* from the pCL1 plasmid formed a white colony-patch indicating that activation of *ADE2* by Gal4 is stronger than by the two STFs identified in this experiment.

Finally using quantitative reverse transcription PCR (qRT-PCR), we measured gene expression levels from *pGAL2*::*ADE2* induced by STFs and by Gal4 and compared the levels to those obtained with *ADE2* in a wild-type strain transformed with the empty plasmid pRS415 (Fig. 5d). STF Cas9-gRNA_46 did not significantly increase *ADE2* expression above background levels (*p* value >0.79); i.e. above levels obtained with a *pGAL2*::*ADE2* reference strain transformed with pRS415. This finding is in agreement with the fact that STF Cas9-gRNA_46 did not support growth on solid SC-Ade-Leu medium (Additional file 1: Figure S7). Also in agreement with the spot assays, the two STFs, Cas9-gRNA_45 and Cas9-gRNA_64, which did support growth on solid SC-Ade-Leu medium, displayed significantly increased *ADE2* transcription, 4.0- and 6.2-fold, respectively (both *p* values <0.005). These levels are approximately 41- and 26-fold lower than the level obtained with Gal4, respectively. The lower *ADE2* mRNA levels obtained with the two STFs may explain the pink colony phenotype. On the other hand, the *ADE2* mRNA levels obtained with the two STFs Cas9-gRNA_45 and Cas9-gRNA_64 from *pGAL2*::*ADE2* are only slightly lower, 2.1- and 1.4-fold, (*p* values <0.005) than the levels measured from a wild-type *ADE2* allele.

#### Using SWITCH in the regulatory state to optimize the naringenin pathway

To investigate whether SWITCH can be used to optimize a metabolic pathway we investigated whether the NG pathway could be optimized by reducing the activity of the essential *TSC13* gene (see Fig. 3). By reducing Tsc13 activity, additional NG is expected as Tsc13 diverts coumaroyl-CoA, an intermediate in the NG pathway, into a competing pathway. Towards this goal, we first used TAPE to identify six efficient *TSC13* specific gRNAs (gRNA_189–gRNA_194) for targeting Cas9 to the *TSC13* ORF (see Additional file 1: Figure S8a and b).

Next, we switched the *cas9* NG strain (S-2) from the genetic engineering state to repressive *dcas9* regulatory state. The resulting *dcas9* NG strain (S-3) was then transformed with each of the *TSC13* interference gRNA plasmids and with pRS415 as negative control. For comparison, a NG reference strain (S-4) that does not contain dCas9 and with restored X-3 locus by SWITCH (Step 3*, Fig. 1), was transformed with the same set of plasmids. From each transformation plate, six clones were cultured in micro-titer dishes using fed-batch medium, where glucose is released by enzymatic hydrolysis of a polysaccharide (see “Methods”).

With all strains, identical final OD_600_ measurements were obtained (*p* values >0.06) indicating that they all contain sufficient Tsc13 activity to sustain wild-type biomass production (Additional file 1: Figure S8c). We then determined the effect of the five *TSC13* specific gRNAs on coumaric acid (COA), NG, and phloretic acid (PHA) production by HPLC-UV/DAD analysis.

**Fig. 6:**
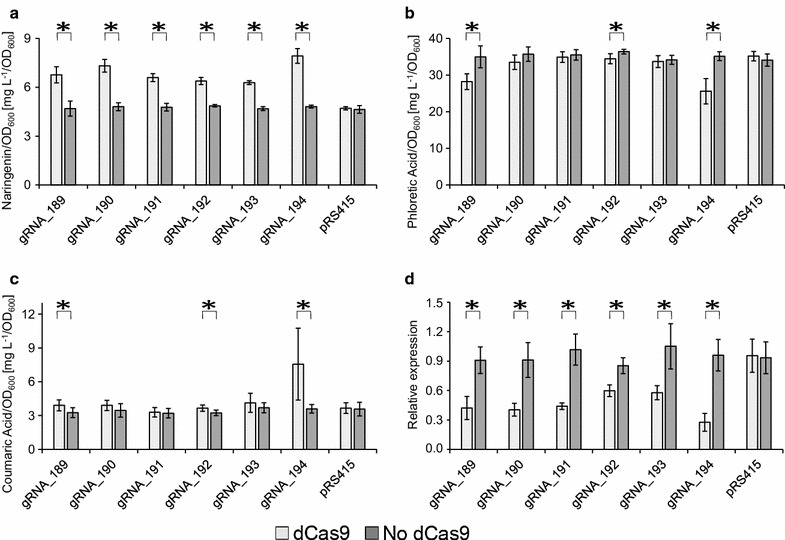
Downregulation of *TSC13* by dCas9 interference. Transformants expressing interference gRNAs were cultured in fed-batch media and HPLC analysis was used to detect: **a** naringenin, **b** phloretic acid and **c** coumaric acid. **d** *TSC13* transcript levels relative to *ACT1* were measured by qRT-PCR. *Stars* indicate significantly different levels of either compounds concentration or *TSC13* expression (*p* values <0.05) in strain pairs containing dCas9 or not, as indicated

With all dCas9-gRNA strains significant increases in NG production, >30%, were observed as compared to NG production in their corresponding reference strains, which did not contain dCas9. The biggest increase, 65%, was obtained with gRNA_194 (Fig. 6a) and this was accompanied by a significant 27% reduction in PHA production (Fig. 6b) indicating that the flux towards this intermediate was reduced. In addition, strains expressing gRNA_194 also accumulated 110% more COA as compared to the reference strain (Fig. 6c). This result suggests that enzymes downstream of this intermediate constitute a significant bottleneck in this strain. Finally, we also measured *TSC13* mRNA levels in this set of strains. In all cases we measured significantly reduced *TSC13* gene activity as compared to the reference strains (Fig. 6d). Importantly, the largest reduction in *TSC13* activity (70%) was observed with the strain expressing gRNA_194, which is the strain producing the highest level of NG. As expected, with the dCas9 strain transformed with pRS415, no changes in COA, NG, PHA production were observed as compared to the corresponding reference strain; and *TSC13* mRNA levels were unchanged.

## Conclusions and perspectives

CRISPR is increasingly used as a genetic engineering tool by exploiting the ability of gRNA-Cas9 to make specific DNA DSBs; and as a gene regulatory tool by exploiting the ability of gRNA-dCas9 to bind specifically to promoter or ORF sequences. For the first time, we have successfully combined these two tools into one system and shown that it can be used to establish and optimize a cell factory. Specifically, the experiments presented above demonstrate that SWITCH can be used to integrate and fine tune a metabolic pathway to increase production yields. Using a multi-step metabolic engineering strategy and a different strain background than ours, Koopman et al. 2012 have reported NG titers of 400 µM (~108 mg/l). Since down-regulation of *TSC13* was not included in their strategy, it could be interesting to investigate whether NG titers could be further increased by combining all the genetic features in one strain. In our proof of concept setup, the gRNA is transcribed from a plasmid as it allowed us to rapidly identify functional variants. A further improvement could therefore be to integrate the gRNA gene in the genome to avoid using selective medium. This would be essential if SWITCH mediated pathway fine tuning is applied to a production strain. In the present study we have regulated expression levels of single genes, but more dramatic effects may be achieved by up- or down-regulating several genes in a metabolic system simultaneously. This principle was recently demonstrated by Cheng et al. 2013 in mammalian cells [31]. Moreover, even more complex regulation may be achieved by including new CRISPR related nucleases [16, 17] with unique gRNA requirements into the SWITCH toolbox. An expanded repertoire of CRISPR nucleases can be exploited in SWITCH for iterative cycles of strain engineering and differential tuning of individual genes or gene sets. Lastly, we envision that SWITCH can be implemented in other species where Cas9 stimulated gene targeting is efficient.

## Methods

### Strains and culture conditions

*Escherichia coli* DB3.1 competent cells from Invitrogen were used as the cloning host for USER vector backbones expressing the *ccdB* gene. Expression vectors were cloned using *E. coli* DH5α competent cells. After transformation, *E. coli* cells were cultured at 37 °C for at least 12 h on Luria broth (LB) plates (1% tryptone, 0.5% yeast extract, 1% NaCl, 2% Agar) supplemented with 100 mg/l ampicillin. Plasmid rescue cultivations were prepared using liquid LB medium with 100 mg/l ampicillin.

Two different *S. cerevisiae* backgrounds were used: S-0 derived from S288C (National Collection of Yeast Cultures, UK, NCYC 3608) and the yeast two hybrid (Y2H) strain PJ69-4 [28]. Genotypic description of all strains can be found in Table 1. Yeast strains were grown on liquid YPD medium (1% yeast extract, 2% peptone, 2% glucose) for transformation. For vector selection after transformation, the strains were cultivated on plates containing synthetic complete (SC) medium minus the corresponding auxotrophic marker (1% succinic acid, 0.6% NaOH, 0.67% yeast nitrogen base without amino acids, 1.5% agar) and supplemented with 2% glucose.

For analysis of NG production, small cultivations were performed using m2p-labs media development kit for glucose-fed batch (M-KIT-100), where glucose is released enzymatically through the cultivation according to the instructions of the manufacturer. The basic components for the fed-batch medium were 2.5% 4× DELFT, 8.55% 0.5 M citrate, 1.145% 1 M K2HPO4, 0.5% milli-q water, 50% concentrated polysaccharide, 8% enzyme mixture, 1% trace metals and 1% vitamins. Small-scale cultivations were carried out in 96 wells microtiter plates at 400 rpm, 30° and 25 mm amplitude or 280 rpm, 30° and 50 mm amplitude. Fed-batch small scale experiments were initiated by inoculating clones into 500 μl SC media lacking leucine. After 12 h incubation the culture was re-inoculated to reach an OD_600_ of 0.1 in a total volume of 500 μl fed-batch media and incubated for 72 h. An EnVision 2104 Plate Reader was used for OD_600_ measurements.

#### Primers, plasmids, rescue fragments and USER cloning

All primers were supplied by IDT and are listed in Additional file 1: Table S1. Plasmids used are listed in Additional file 1: Table S2.

USER cloning was used to construct integration and centromere expression plasmids [32]. PCR USER compatible fragments were amplify using PfuX7 polymerase [33]. Backbone plasmids were digested with *AsiSI* and *Nb.BsmI* (New England Biolabs, 1 U/μl) to remove the *ccdB* gene and create USER compatible ends. Equimolar amounts of purified PCR products and pre-digested backbone were mixed to reach a final volume of 8 µl. Finally, 1 μl of USER™ enzyme mix (New England Biolabs, 1 U/μl) and 1 μl of 10 × Standard Taq Reaction Buffer (10 mM Tris–HCl, 50 mM KCl, and 1.5 mM MgCl_2_, pH 8.3) were added. The final reaction mixture was incubated for 20 min at 37 °C, followed by 20 min at 25 °C. The treated USER mixture was then used to transform 50 µl of chemically competent *E. coli* cells by heat shock.

To generate the different types of *cas9* gene fragments for integration into the X-3 locus, the human codon optimized *cas9* gene from Addgene plasmid #43802 and the yeast codon optimized *dcas9* from Addgene plasmid #64279, were PCR amplified using USER compatible primers. The PCR amplified *cas9* and *dcas9* fragment were each cloned into the single integration plasmid X-3 together with a PCR amplified USER compatible TEF1 promoter [20]. Fused *dcas9* with VP64 was generated by ordering a VP64 gblock from IDT which was PCR amplified using USER compatible primers. The PCR amplified TEF1 promoter, *dcas9* and VP64 were USER cloned into X-3 single integration plasmid.

A compatible uracil excision-based cloning cassette*, AsiSI*/*Nb.BsmI*-*ccdB*-*AsiSI/Nb.BsmI* was USER cloned into a USER compatible pRS415 [34] backbone to create plasmid pKGV4227. gRNA genes and pSNR52 USER compatible fragments were PCR amplified from Addgene plasmid #43803 and were clone into pre-digested pKGV4227, with *AsiSI* and *Nb.BsmI*.

Rescue fragments encoding either *dcas9* or *dcas9*-VP64 were PCR amplified from plasmids pKGV7 and pKGV5 respectively, using forward primers (FW) with homology to *cas9* at the 5′ end and reverse primers (RV) with sequence homologous to the nuclear localization signal (NLS) and *tCYC1* at the 3′ end. A rescue fragment for X-3 locus restoration was amplified using genomic DNA extracted from s288c.

NG pathway genes were codon optimized for *S. cerevisiae* by GeneArt and assembled into three different constructs that were used for HR into the XI-2 locus. Each assembler construct was designed to express two genes by divergently oriented promoters, which gives the possibility to integrate up to six genes at one integration site. In this study, the first assembler construct expresses the Phenylalanine ammonia-lyase 2 (Pal2), under the control of *pTDH3* and the Cinnamate-4-hydroxylase linked to the NADPH-cytochrome P450 reductase 2 (C4H-ATR2), under the control of *pTEF2*. All genes originated from *Arabidopsis thaliana* (At). Assembler-two construct contains the *Petunia hybrida* chalcone-flavonone isomerase A (*Ph*CHI), under the control of *pTEF1* and the *Hypericum androsaemum* naringenin-chalcone synthase (*Ha*CHS), under the control of *pPGK1*. In assembler three 4-coumarate:coenzyme A ligase 2 (*At*4CL2) was express by *pPDC1*. Assembler one was flanked by XI-2 locus DOWN HR and by the divergently oriented terminator construct *tFBA1*-*tPGl1*. The assembler two construct was flanked by two divergently oriented terminators construct *tFBA1*-*tPGl1* and *tTDH2*-*tENO2*. The assembler three construct was flanked by the divergently oriented terminator construct *tTDH2*-*tENO2* and by XI-2 locus UP HR. The assembler fragments were integrated via HR by the divergently oriented terminators with each other and with the locus XI-2 by the UP and DOWN HR. All PCR results were evaluated using Thermo Scientific O’GeneRuler 1 kb DNA Ladder.

### Transformation

*S. cerevisiae* strains were transformed with different combinations of either linearized fragments for chromosomal integration or with centromere plasmids by the lithium acetate transformation method [35]. Prior to transformation, integrative plasmids were digested with *NotI* (New England Biolabs, 1 U/μl), 400 ng of digested plasmid was used for each transformation. For centromere plasmids, 100 ng DNA was used per transformation. Integration of linearized fragments was verified by yeast colony PCR, where single colonies were picked from transformation plates and pre-treated by boiling in a microwave oven at 900 watts for 1 min. Amplification was performed using Thermo Scientific DreamTaq DNA Polymerase.

### Sample preparation and analytical methods

After 72 h of cultivation, OD_600_ of fed-batch cultures was measured using an EnVision 2104 Plate Reader. HPLC samples were prepared by extracting the supernatant after the culture was diluted 2 times with 96% ethanol. HPLC analysis was performed using Thermo Scientific Dionex Ultimate3000 equipped with Ultra C18 3 µm Column (100*4.6 mm). The gradient method mobile phase was composed of two solvents, water (A) and acetonitrile (B), both buffered with 0.05% trifluoroacetic acid (TFA). The column temperature was maintained at 40 °C and the flow rate was kept at 1 ml/min. A short program was developed for quick screening of NG production, where the fraction of solvent B was first increased linearly from 15 to 35% (0–3.2 min) and subsequently from 35 to 100% (3.2–3.5 min). The B fraction remained at 100% until the end of the program (3.5–4 min). Samples used to measure NG and intermediates COA and PHA were analyzed using a longer gradient program. Fraction B was increased linearly from 20 to 30% (0–4.5 min) and afterwards from 30 to 60% (4.5–9 min). The B fraction remained at 60% until the end of the program (9–9.1 min). In both programs the initial gradient conditions were re-set by an equilibrium program (−3 to 0 min). The peak area of the compounds was integrated and used for quantification by fitting with a standard curve.

### qRT-PCR analysis

Yeast RNA was extracted using the RiboPure™-Yeast Kit (Thermo Fisher Scientific). Approximately 200 ng of RNA was used with the AccuScript High Fidelity 1st Strand cDNA Synthesis Kit (Agilent Technologies) and 5 μl of cDNA was used for each qPCR reaction, utilizing the SYBR^®^ Select Master Mix. qRT-PCR primers are listed in Additional file 1: Table S1. qRT-PCR was run and analysed on the Mx3005P QPCR System (Stratagene), all expression levels were normalized to *ACT1* mRNA levels.

### Statistics

All TAPE values were based on two independent transformations using the same batch of competent yeast cells. All relative expression qRT-PCR values were derived from single colonies, which were analysed by qRT-PCR in technical triplicates. All small scale cultivations for heterologous pathway evaluation were performed as biological sextuplicates. In all cases, two-tailed two-sample *t* tests were performed to evaluate whether two average values in a specified experimental set were significantly different.

## Additional file

**Additional file 1: Figure S1.** The SWITCH recombination event triggering substitution of *cas9* (human codon optimized) for *dcas9* (yeast codon optimized). **Figure S2**. Confirmation of *cas9* integration into the X-3 locus by diagnostic PCR. **Figure S3**. Control screening in strain S-0 of the three gRNAs tested for swapping *cas9*. **Figure S4**. Exploiting SWITCH for marker-free integration into a specific locus. **Figure S5**. The three assembler fragments used with SWITCH for marker-free integration of the complete naringenin pathway into locus XI-2. **Figure S6**. Naringenin producers created by SWITCH and assembler. **Figure S7**. Gene regulation by SWITCH. **Figure S8**. Implementing SWITCH for *TSC13* down regulation. **Table S1**. The main primers used in this study. **Table S2**. Plasmid list.
