# Erratum to: SWITCH: a dynamic CRISPR tool for genome engineering and metabolic pathway control for cell factory construction in *Saccharomyces cerevisiae*

**DOI:** 10.1186/s12934-017-0668-y

**Published:** 2017-03-28

**Authors:** Katherina García Vanegas, Beata Joanna Lehka, Uffe Hasbro Mortensen

**Affiliations:** 1 Department of Biotechnology and Biomedicine, Technical University of Denmark, Søltofts Plads, Building 223, Room 208, 2800 Kgs. Lyngby, Copenhagen, Denmark; 2 Department of Science and Environment, Roskilde University, Universitetsvej 1, 4000 Roskilde, Denmark

## Erratum to: Microb Cell Fact (2017) 16:25 DOI 10.1186/s12934-017-0632-x

Upon publication of this article [1], it was brought to our attention that Figure 3 contained the following errors:

1. The figure shows the pathway towards Naringenin in which a single hydroxyl group is positioned incorrectly in Naringenin chalcone, Naringenin and Phloretin, because the bond connecting the right side ring system was accidentally placed towards carbon number 6 instead of carbon number 1.
2. In addition, the structures of Cinnamic acid and Coumaric acid are shown as their corresponding bases.

The above errors do not influence the science and conclusions presented in the article [1].
