# Supplementary material for: SWITCH: a dynamic CRISPR tool for genome engineering and metabolic pathway control for cell factory construction in Saccharomyces cerevisiae
Source: Microb Cell Fact. 2017 Feb 8;16:25. doi: 10.1186/s12934-017-0632-x (PMC5299646; doi:10.1186/s12934-017-0632-x)
Supplement: Supplementary file 1 — Additional file 1: Figure S1. The SWITCH recombination event triggering substitution of cas9 (human codon optimized) for dcas9 (yeast codon optimized). Figure S2. Confirmation of cas9 integration into the X-3 locus by diagnostic PCR. Figure S3. Control screening in strain S-0 of the three gRNAs tested for swapping cas9. Figure S4. Exploiting SWITCH for marker-free integration into a specific locus. Figure S5. The three assembler fragments used with SWITCH for marker-free integration of the complete naringenin pathway into locus XI-2. Figure S6. Naringenin producers created by SWITCH and assembler. Figure S7. Gene regulation by SWITCH. Figure S8. Implementing SWITCH for TSC13 down regulation. Table S1. The main primers used in this study. Table S2. Plasmid list. [file 12934_2017_632_MOESM1_ESM.docx]

# SWITCH - a dynamic CRISPR tool for genome engineering and metabolic pathway control for cell factory construction in *Saccharomyces cerevisiae*.

Katherina García Vanegas^1^, Beata Joanna Lehka ^2^, Uffe Hasbro Mortensen^1^*

**Email addresses:**

KGV: kgava@bio.dtu.dk

BJL: blehka@ruc.dk

UHM: um@bio.dtu.dk

^1^Department of Biotechnology and Biomedicine, Technical University of Denmark, Søltofts Plads, DK-2800, Kgs. Lyngby, Denmark

^2^Department of Science and Environment, Roskilde University, Universitetsvej 1, DK-4000, Roskilde, Denmark

 * Corresponding author

# Additional file 1

## Figures


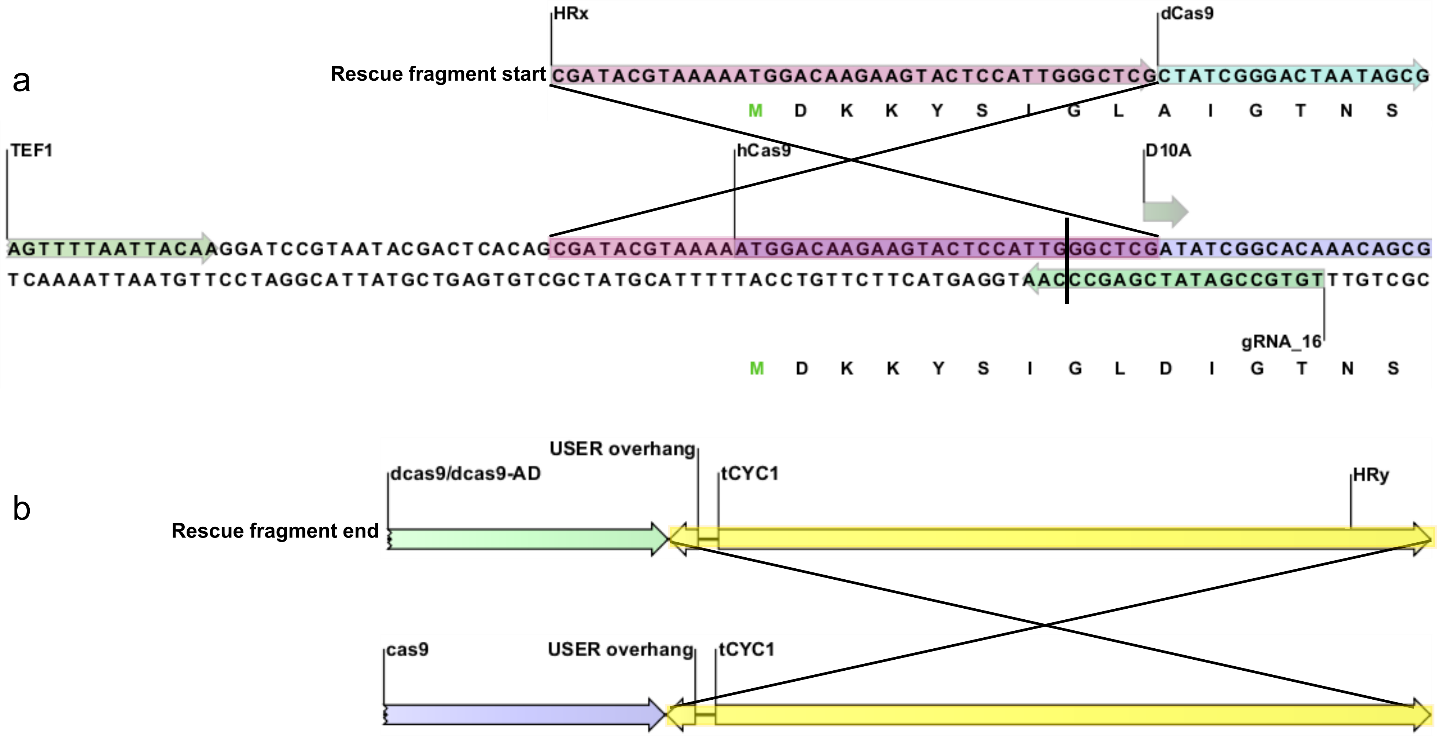


Figure S1: The SWITCH recombination event triggering substitution of *cas9* (human codon optimized) for *dcas9* (yeast codon optimized). The upstream section of the rescue fragment (a) contains a targeting sequence composed by the first 28 bp of *cas9* (which are identical to *dcas9*) and 12 bp of the linker region between *pTEF1* and *cas9*. The end region (b) contains 190 bp of *tCYC1* and 13 bp of the USER overhang. Hence, when this fragment serves as a repair template for the Cas9 induced DSB in *cas9*, the genetic information encoding *dcas9* integrates via HR into the specific locus at the expense of the Cas9 gene.


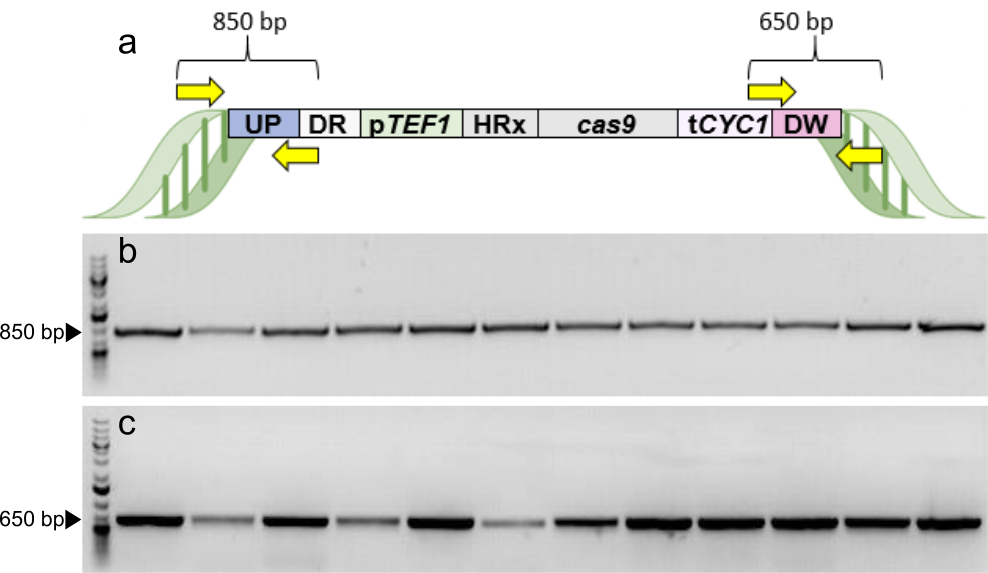


Figure S2: Confirmation of *cas9* integration into the X-3 locus by diagnostic PCR. (a) A schematic representation of the X-3 locus containing *cas9*. The position of primers for the diagnostic PCR reactions are indicated.12 randomly selected clones from SC-Ura plates were tested. Diagnostic PCR fragments for the UP (b) and the DOWN (DW) (c) integration region are 850 and 650 bp, respectively.


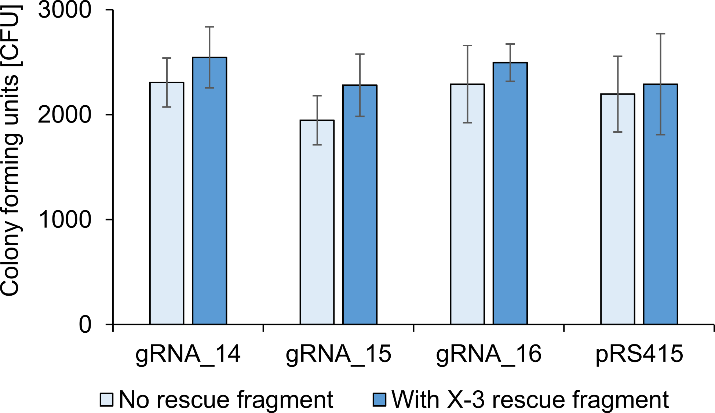


Figure S3: Control screening in strain S-0 of the three gRNAs tested for swapping *cas9*.


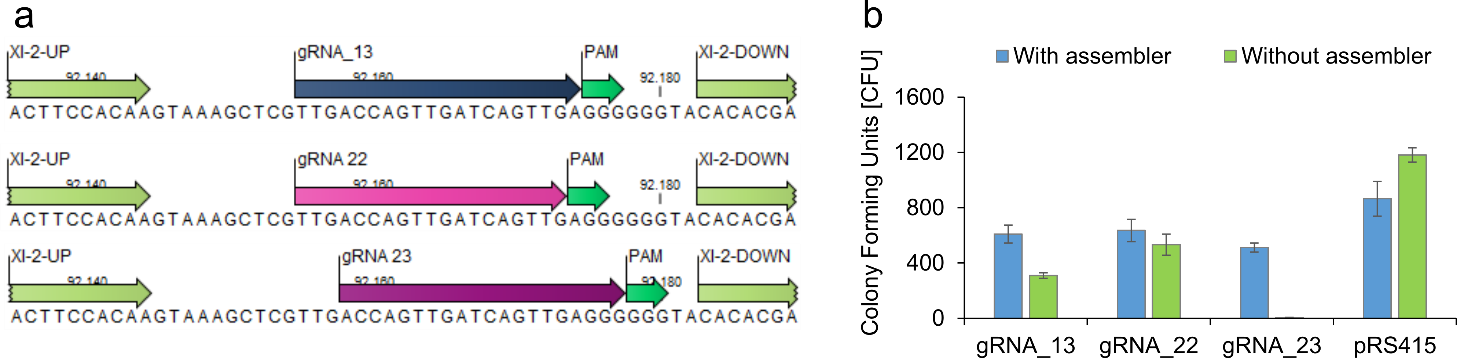


Figure S4: Exploiting SWITCH for marker-free integration into a specific locus. (a) Localization of the three gRNAs tested for marker-free integration into the XI-2 locus. (b) Screening of the three gRNAs efficiency to guide Cas9 between the UP and DOWN integration regions in locus XI-2 using TAPE.


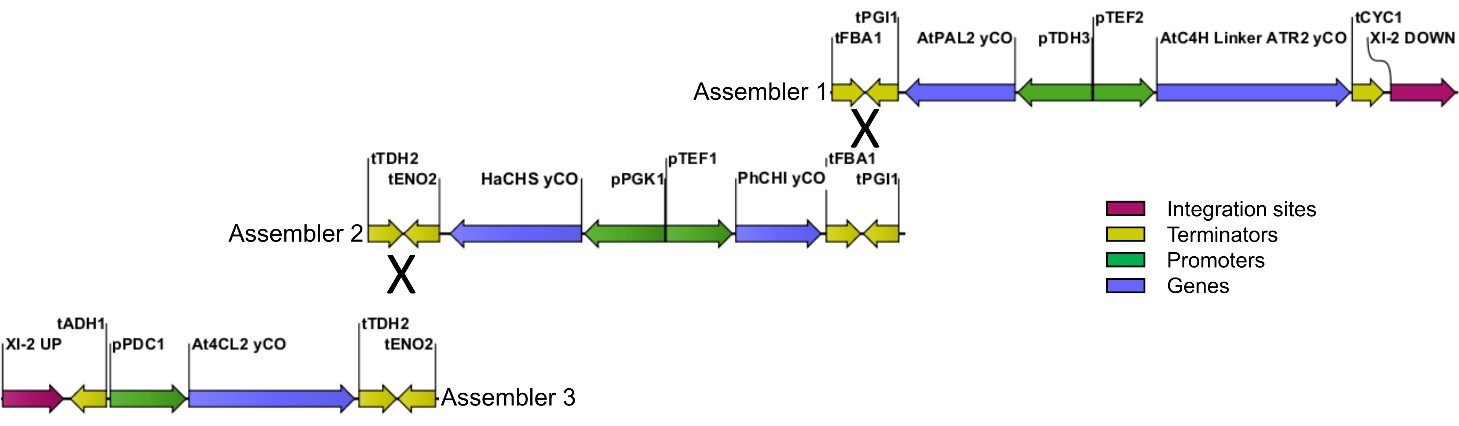


Figure S5: The three-assembler fragments used with SWITCH for marker-free integration of the complete naringenin pathway into locus XI-2. All genes were codon optimized for yeast codon usage (yCO).


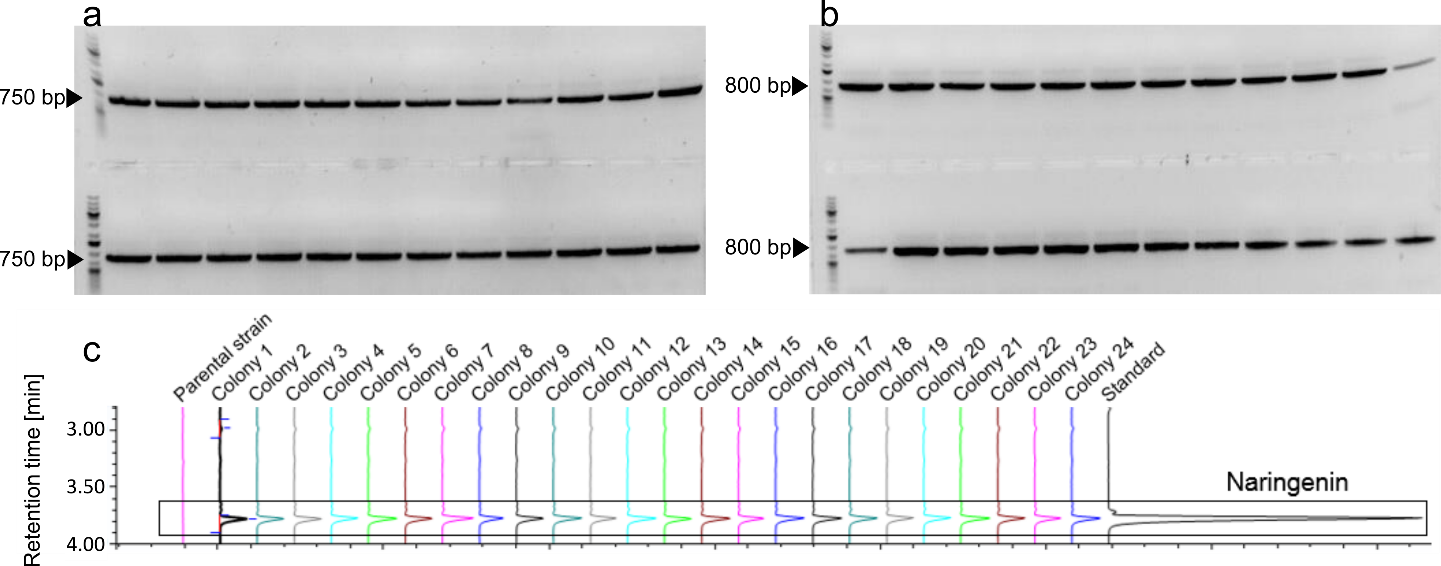


Figure S6: Naringenin producers created by SWITCH and assembler. Diagnostic PCR of 24 clones randomly selected after co-transformation with gRNA_23 and three assembler fragments. Diagnostic PCR for validating the UP region (a) and the DOWN region (b) at the XI-2 locus. (c) All 24 clones were analyzed by HPLC analysis UV/DAD to validate naringenin production. The concentration of the naringenin standard was 250 mg L^-1^.


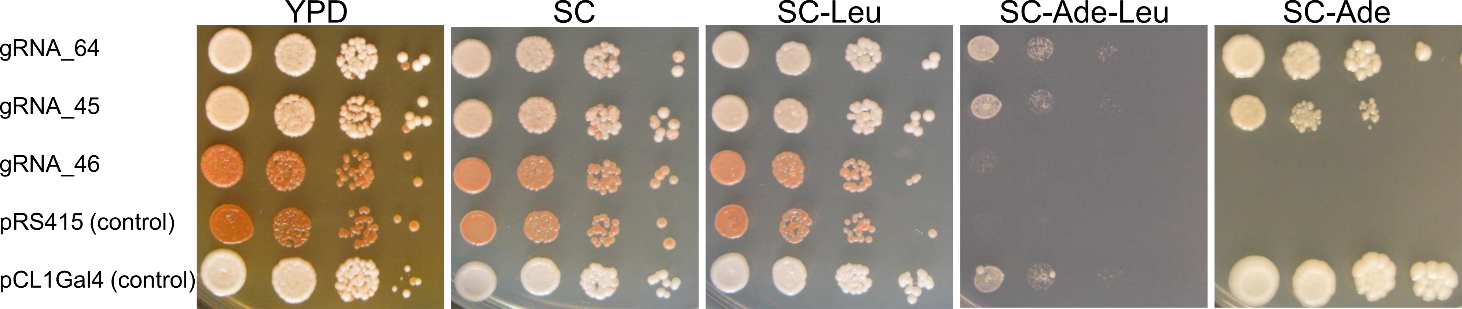


Figure S7: Gene regulation by SWITCH. Use of dCas9-VP64 as a synthetic transcription factor for activation of *ADE2* in a two-hybrid strain background, see text for details. Cultures of PJ69-4 S-2 co-transformed with different gRNAs and control plasmids were 10-fold serial diluted and analyzed in a spot test using different types of solid media as indicated.


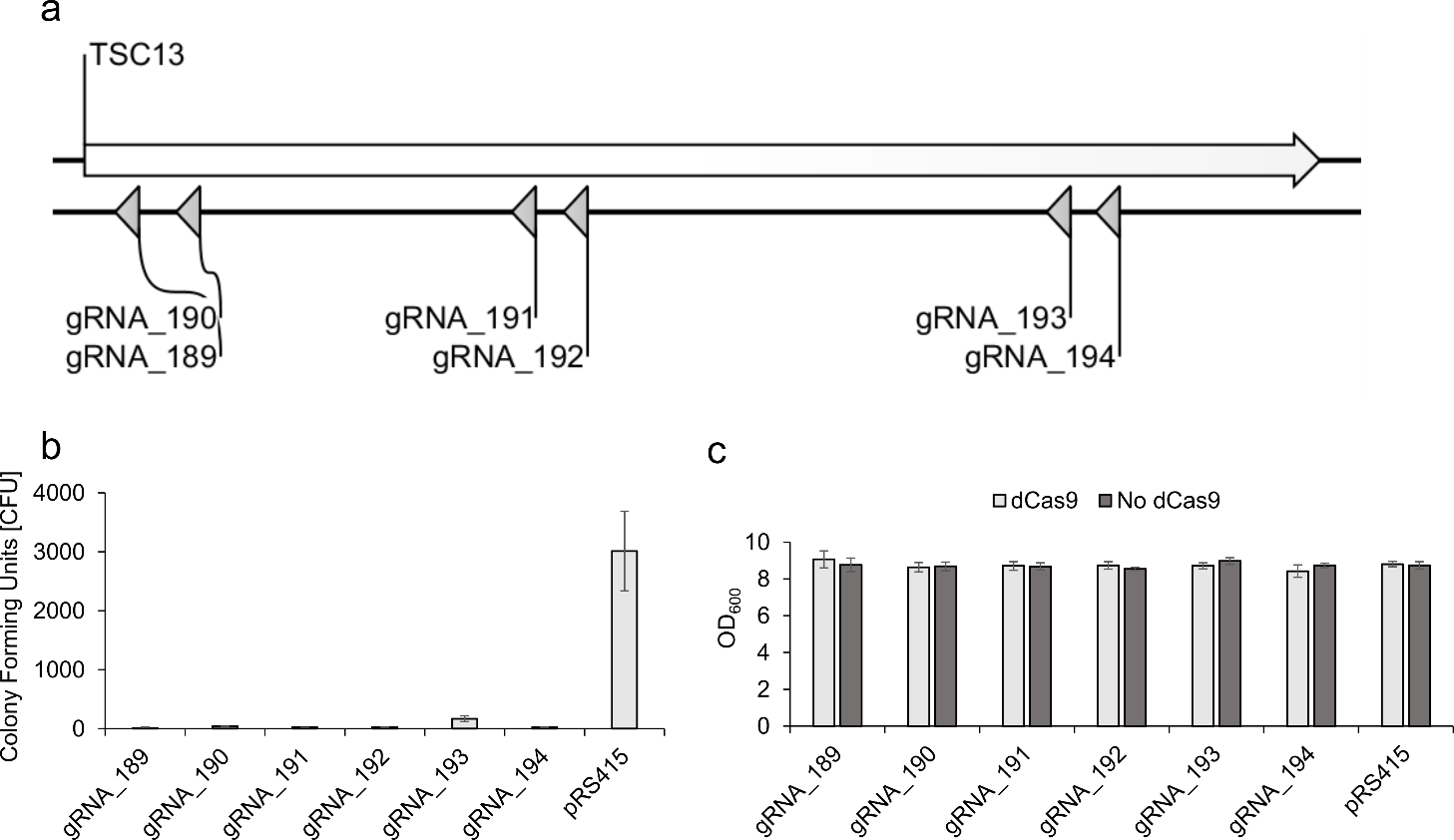


**Figure S8:** Implementing SWITCH for *TSC13* down regulation. **(a)** Localization of the six gRNAs tested for downregulation of *TSC13* **(b)** Screening of the efficiency of the six gRNAs to guide Cas9 to the *TSC13* gene using TAPE**.** All numbers of transformants obtained with gRNA encoding plasmids were significantly lower (all p-values < 0.05) than the numbers obtained with the control vector pRS415. **(c)** Cultures final OD_600_ measurements performed after 72 hours incubation, t-test confirmed that there were no significant differences (all p-values > 0.06) between dCas9 strains and their respective reference strains.

## Tables

Table S1: The main primers used in this study

| **Primer** | **Primer sequence, 5´ to 3´** | **Purpose** |
| --- | --- | --- |
| pTEF FW USER | CGTGCGAUGCCGCACACACCATAGCTT | Primers for cloning |
| pTEF RV USER | ACGTATCGCUGTGAGTCGTATTACGGATCCTTG | Primers for cloning |
| hCas9 FW USER | AGCGATACGUAAAAATGGACAAGAAGTACTCCA | Primers for cloning |
| hCas9 RV USER | CACGCGAUTCACACCTTCCTCTTCTTCTT | Primers for cloning |
| dCas9 FW USER | AGCGATACGUAAAAATGGAATTAGATCTCGCCAC | Primers for cloning |
| dCas9 RV USER | CACGCGAUCTAGGATCCGGAACTACC | Primers for cloning |
| dCas9 RV VP64 USER | AGCGGAACUACCTACCTTGCGCTTTTTCTTG | Primers for cloning |
| VP64 FW USER | AGTTCCGCUGACGCATTGGACGATTTT | Primers for cloning |
| VP64 RV USER | CACGCGAUCTACAGCATGTCCAGGTCGAAA | Primers for cloning |
| Integration vector check UP | TCTCAGGTATAGCATGAGGTCGCTCAT | Primers for verification |
| Integration vector check DW | CCTGCAGGACTAGTGCTGAGGCATTAAT | Primers for verification |
| X-3 check UP | TGACGAATCGTTAGGCACAG | Primers for verification |
| X-3 check DW | CCGTGCAATACCAAAATCGAG | Primers for verification |
| Donor X-3  FW | CGAGATCTTTGTGTTCGGTTAC | Primers for rescue fragment amplification |
| Donor X-3  RV | GGCATTAAAAACAAACGA | Primers for rescue fragment amplification |
| Donor dCas9±RD FW | CGATACGTAAAAATGGACAAGAAGTACTCCATTGGGCTCGCTATCGGGACTAATAGCGTC | Primers for rescue fragment amplification |
| Donor dCas9±RD RV | CTTCGAGCGTCCCAAAACCT | Primers for rescue fragment amplification |
| SWITCH check FW | TTGCAAGGAAGAAGGATTG | Primers for verification |
| SWITCH check RV | CTCCTTCCTTTTCGGTTA | Primers for verification |
| XI-2 check UP | GTTTGTAGTTGGCGGTGGAG | Primers for verification |
| XI-2 check DW | GAGACAAGATGGGGCAAGAC | Primers for verification |
| pTEF1/pPGK1 FW | ACGTATCGCUGTGAGTCGTATTACGGATCC | Primers for cloning |
| pTEF1/pPGK1 RV | ACCCGTTGAUGCCGCTTGTTTTATATTTGTTGTAAAAAG | Primers for cloning |
| *pTEF2/pTDH3 FW* | ACCCGTTGAUTTTTGTTTGTTTATGTGTG | Primers for cloning |
| *pTEF2/pTDH3 RV* | ACGTATCGCUTGTTTAGTTAATTATAGTTC | Primers for cloning |
| *pPDC1 FW* | CGTGCGAUGCCGATCTATGCGACTGGGTGAG | Primers for cloning |
| *pPDC1 RV* | ACGTATCGCUTTTTGATAGATTTGACTGTGTTATTTTGCG | Primers for cloning |
| *AtC4H FW* | AGCGATACGUAAAATGGATTTGTTATTGCTGGAAAAG | Primers for cloning |
| *AtC4H L5 RV* | AGCTGCAGCUTCTTTTGCTGCAGCTTCAGCGCTACAATTTCTGGGTTTCATG | Primers for cloning |
| *ATR2 L5 FW* | AGCTGCAGCUAAAGAAGCTGCAGCAAAAGC | Primers for cloning |
| *ATR2 RV* | CACGCGAUTTACCATACATCTCTCAGATATCTAC | Primers for cloni |
| *AtPAL2 FW* | ATCAACGGGUAAAATGGACCAAATTGAAGCAATGC | Primers for cloning |
| *AtPAL2 RV* | CGTGCGAUTTAGCAGATTGGAATAGGTGCAC | Primers for cloning |
| *At4CL2 FW* | AGCGATACGUAAAATGACGACACAAGATGTGATAGTC | Primers for cloning |
| *At4CL2 RV* | CACGCGAUCTAGTTCATTAATCCATTTGCTAG | Primers for cloning |
| *HaCHS FW* | ATCAACGGGUAAAATGGTTACTGTTGAAGAAGTTAG | Primers for cloning |
| *HaCHS RV* | CGTGCGAUTAATTAATTGCGACTGAATGAAG | Primers for cloning |
| *PhCHI FW* | AGCGATACGUAAAATGTCTCCACCAGTTTCTGTTAC | Primers for cloning |
| *PhCHI RV* | CACGCGAUCTACACACCGATAACAGGTATTG | Primers for cloning |
| pRS415 FW USER *ccdB* | AGCGATCGCGTGCATUCATCCGCTCTAACCGAAAAG | Primers for cloning |
| pRS415 RV USER *ccdB* | AGCGATCGCACGCATUCTTTAGTGAGGGTTAATTG | Primers for cloning |
| *ccdB* USER FW | AATGCGTGCGATCGCUAGCCTACTCGCTATTGTCCTCA | Primers for cloning |
| *ccdB* USER RV | AATGCACGCGATCGCUGCGCCGAATAAATACCTGT | Primers for cloning |
| SNR52p FW | CGTGCGAUTCTTTGAAAAGATAATGTATG | Primers for cloning |
| SNR52p RV | ATCATTTAUCTTTCACTGCGGA | Primers for cloning |
| gRNA RV | CACGCGAUAGACATAAAAAACAAAAAAAGC | Primers for cloning |
| gRNA_14 for swap FW | ATAAATGAUCATGGACAAGAAGTACTCCATGTTTTAGAGCTAGAAATAGCA | Primers for cloning |
| gRNA_15 for swap FW | ATAAATGAUCTACTCCATTGGGCTCGATATGTTTTAGAGCTAGAAATAGCA | Primers for cloning |
| gRNA_16 for swap FW | ATAAATGAUCTGTGCCGATATCGAGCCCAAGTTTTAGAGCTAGAAATAGCA | Primers for cloning |
| gRNA_13 for ChrXI-2 FW | ATAAATGAUCTTGACCAGTTGATCAGTTGAGTTTTAGAGCTAGAAATAGCA | Primers for cloning |
| gRNA_22 for ChrXI-2 FW | ATAAATGAUCTTGACCAGTTGATCAGTTGAGTTTTAGAGCTAGAAATAGCA | Primers for cloning |
| gRNA_23 for ChrXI-2 FW | ATAAATGAUCACCAGTTGATCAGTTGAGGGGTTTTAGAGCTAGAAATAGCA | Primers for cloning |
| gRNA_38 for regulation of Gal2p FW | ATAAATGAUCAAGATTCTACCGCATTTAATGTTTTAGAGCTAGAAATAGCA | Primers for cloning |
| gRNA_39 for regulation of Gal2p FW | ATAAATGAUCAAAGGTACTCAACGTCAATTGTTTTAGAGCTAGAAATAGCA | Primers for cloning |
| gRNA_40 for regulation of Gal2p FW | ATAAATGAUCGCAACCTACTTAAGCCATTCGTTTTAGAGCTAGAAATAGCA | Primers for cloning |
| gRNA_41 for regulation of Gal2p FW | ATAAATGAUCTTCTATTAGTAGCTAAAAATGTTTTAGAGCTAGAAATAGCA | Primers for cloning |
| gRNA_42 for regulation of Gal2p FW | ATAAATGAUCAAGGCACATGGACCCCTGAAGTTTTAGAGCTAGAAATAGCA | Primers for cloning |
| gRNA_43 for regulation of Gal2p FW | ATAAATGAUCTTAAGGCAGAAGGCAGTATCGTTTAGAGCTAGAAATAGCA | Primers for cloning |
| gRNA_44 for regulation of Gal2p FW | ATAAATGAUCGGGCTTAACTAATCTCGGTTGTTTTAGAGCTAGAAATAGCA | Primers for cloning |
| gRNA_45 for regulation of Gal2p FW | ATAAATGAUCATCTCAAGATGGGGAGCAAAGTTTTAGAGCTAGAAATAGCA | Primers for cloning |
| gRNA_46 for regulation of Gal2p FW | ATAAATGAUCTGCACAGTTAACTTTCTAGCGTTTTAGAGCTAGAAATAGCA | Primers for cloning |
| gRNA_47 for regulation of Gal2p FW | ATAAATGAUCTCTTAAATTATACAACATTCGTTTTAGAGCTAGAAATAGCA | Primers for cloning |
| gRNA_48 for regulation of Gal2p FW | ATAAATGAUCTTCTTGATGATAATTGAATAGTTTTAGAGCTAGAAATAGCA | Primers for cloning |
| gRNA_49 for regulation of Gal2p FW | ATAAATGAUCCAGTAATTGGATTGAAAATTGTTTTAGAGCTAGAAATAGCA | Primers for cloning |
| gRNA_50 for regulation of Gal2p FW | ATAAATGAUCAAAAAATAATTCTTTCATAAGTTTTAGAGCTAGAAATAGCA | Primers for cloning |
| gRNA_51 for regulation of Gal2p FW | ATAAATGAUCACATTTCGCAGGCTAAAATGGTTTTAGAGCTAGAAATAGCA | Primers for cloning |
| gRNA_64 for regulation of Gal2p FW | ATAAATGAUCGCCCTTCCCATCTCAAGATGGTTTTAGAGCTAGAAATAGCA | Primers for cloning |
| gRNA_189 for regulation of TSC13 FW | ATAAATGAUCTGTCCCTTAACCCTTTAGAGGTTTTAGAGCTAGAAATAGCA | Primers for cloning |
| gRNA_190 for regulation of TSC13 FW | ATAAATGAUCTTCAAAACATCATCTAAAGTGTTTTAGAGCTAGAAATAGCA | Primers for cloning |
| gRNA_191 for regulation of TSC13 FW | ATAAATGAUCTCTATCAACAACTGTGGGAAGTTTTAGAGCTAGAAATAGCA | Primers for cloning |
| gRNA_192 for regulation of TSC13 FW | ATAAATGAUCGTTTAAAAATGGATTATAGTGTTTTAGAGCTAGAAATAGCA | Primers for cloning |
| gRNA_193 for regulation of TSC13 FW | ATAAATGAUCTGAAAATACCTTGATTCAATGTTTTAGAGCTAGAAATAGCA | Primers for cloning |
| gRNA_194 for regulation of TSC13 FW | ATAAATGAUCCAAACTTCAAAAGTATAGTTGTTTTAGAGCTAGAAATAGCA | Primers for cloning |
| *ACT1 FW* | ACTGAAGCTCCAATGAACCCTA | Primers for qRT-PCR |
| *ACT1 RV* | GTCCAAGGCGACGTAACATAGT | Primers for qRT-PCR |
| *ADE2 FW* | TGGACTTGGAAGCAATGG | Primers for qRT-PCR |
| *ADE2 RV* | TAACTTGAATCATAAGCGCC | Primers for qRT-PCR |
| *TSC13 FW* | ACTGGGTTCTAAGCGGTCTC | Primers for qRT-PCR |
| *TSC13 RV* | AACTTAGCATTCCCAAAGGG | Primers for qRT-PCR |

Table S2: Plasmid list

| **Plasmid ID** | **Type** | **Insert** | **Source** |
| --- | --- | --- | --- |
| pRS415 | Centromeric plasmid | *LEU2*, *Amp^r^* | [34] |
| pCL1 | Centromeric plasmid | *LEU2, Amp^r^, GAL4* (1-881) | [30] |
| pCfB127 | Single integration backbone | [pX-3-USER cassette -URA3-DR] , *Amp^r^* | [19] |
| pKGV3 | Single integration | *[X-3::DR-URA3/pTEF1-hCas9-tCYC1] Amp^r^* | *This study* |
| pKGV7 | Single integration | *[X-3::DR-URA3/pTEF1-dCas9_yco-tCYC1] Amp^r^* | *This study* |
| pKGV5 | Single integration | *[X-3::DR-URA3/pTEF1-dCas9-VP64_yco-tCYC1] Amp^r^* | *This study* |
| pKGV4227 | Centromeric plasmid backbone | USER cassette*, LEU2, Amp^r^* | *This study* |
| p190 | Assembler 1 backbone | [pXI-2 DOWN- USER cassette- *tPGI1-tFBA1*], *Amp^r^* | *This study* |
| p858 | Assembler 2 backbone | [*tENO2-tTDH2*-USER cassette- *tPGI1-tFBA1*], *Amp^r^* | *This study* |
| p191 | Assembler 3 backbone | [*tENO2-tTDH2*-USER cassette -*XI-2 UP*], *Amp^r^* | *This study* |
| pAs1 | Assembler 1 integration | *XI-2 DOWN-[tCYC1-AtC4H-L5-ATR2_yco-pTEF2-pTDH3-AtPAL2_yco-tPGI1]-tFBA1, Amp^r^* | *This study* |
| pAs2 | Assembler 2 integration | *tPGl1-[tFBA1-PhCHI_yco-pTEF1-pPGK1-HaCHS_yco-tENO2]-tTDH2, Amp^r^* | *This study* |
| pAs3 | Assembler 3 integration | *tENO2-[tTDH2-At4CL2-pPDC1]-XI-2 UP, Amp^r^* | *This study* |
